# Hemorrhage from the Uterus

**Published:** 1894-02

**Authors:** 


HEMORRHAGE FROM THE UTERUS.—Dr. Savage (*Med. Press and Circ.*, Vol. LV., No. 2811). Uterine hemorrhage may be divided into two classes: 1. That associated directly with the uterus itself. 2. That associated with some condition outside the uterus. Under the first group are included chronic metritis, chronic endometritis, fungous endometritis, subinvolution, incomplete abortion, carcinoma, epithelioma and myomata. Under the second heading are cystomata, extra-uterine pregnancy and chronic inflammatory diseases of the appendages. In this class are also included obesity, constipation and some diseases of the heart and liver. The treatment for uterine hemorrhage varies from the cause. For diseases of the endometrium, the local application of strong carbolic acid or fuming nitric acid, thermo or actual cautery, the positive or acid pole of a galvanic battery and curetting are the usual remedies. In incomplete abortion, retained products of conception should be removed by the aid of forceps or curette. In hemorrhage from cancer of the cervix active methods are advisable; either total extirpation, if practicable, or the removal of as much of the diseased organ as possible by means of the curette or cautery, in this way often prolonging life several months. For myomata, ligation of the ovarian arteries, or the total extirpation of the organ is indicated. The use of corrosive application, at least any stronger than carbolic acid, for disease of endometrium, is to be deprecated.—*The Med. Progress.*

